# A novel p.Gly603Arg mutation in *CACNA1F* causes Åland island eye disease and incomplete congenital stationary night blindness phenotypes in a family

**Published:** 2011-12-15

**Authors:** Ajoy Vincent, Tom Wright, Megan A. Day, Carol A. Westall, Elise Héon

**Affiliations:** Department of Ophthalmology and Vision Sciences, The Hospital for Sick Children, University of Toronto, Toronto, Canada

## Abstract

**Purpose:**

To report, for the first time, that X-linked incomplete congenital stationary night blindness (CSNB2A) and Åland island eye disease (AIED) phenotypes coexist in a molecularly confirmed pedigree and to present novel phenotypic characteristics of calcium channel alpha-1F subunit gene (*CACNA1F*)-related disease.

**Methods:**

Two affected subjects (the proband and his maternal grandfather) and an unaffected obligate carrier (the proband’s mother) underwent detailed ophthalmological evaluation, fundus autofluorescence imaging, and spectral-domain optical coherence tomography. Goldmann visual field assessment and full-field electroretinogram (ERG) were performed in the two affected subjects, and multichannel flash visual evoked potential was performed on the proband. Scotopic 15 Hz flicker ERG series were performed in both affected subjects to evaluate the function of the slow and fast rod pathways. Haplotype analysis using polymorphic microsatellite markers flanking *CACNA1F* was performed in all three family members. The proband’s DNA was sequenced for mutations in the coding sequence of *CACNA1F* and nyctalopin (*NYX*) genes. Segregation analysis was performed in the family.

**Results:**

Both affected subjects had symptoms of nonprogressive nyctalopia since childhood, while the proband also had photophobia. Both cases had a distance visual acuity of 20/50 or better in each eye, normal contrast sensitivity, and an incomplete type of Schubert-Bornschein ERGs. The proband also had high myopia, a mild red-green color deficit, hypopigmented fundus, and foveal hypoplasia with no evidence of chiasmal misrouting. Spectral-domain optical coherence tomography confirmed the presence of foveal hypoplasia in the proband. The clinical phenotype of the proband and his maternal grandfather fit the clinical description of AIED and CSNB2A, respectively. The fundus autofluorescence and the visual fields were normal in both cases; the scotopic 15 Hz flicker ERG demonstrated only fast rod pathway activity in both. Both affected cases shared the same haplotype across *CACNA1F*. The proband carried a novel hemizygous c.1807G>C mutation (p.G603R) in the *CACNA1F* gene. The change segregated with the disease phenotypes and was not identified in 360 control chromosomes. No mutations were identified in *NYX.*

**Conclusions:**

This report of a missense mutation in *CACNA1F* causing AIED and CSNB2A phenotypes in a family confirms that both diseases are allelic and that other genetic or environmental modifiers influence the expression of *CACNA1F*. This is the first report to suggest that in *CACNA1F-*related disease, the rod system activity is predominantly from the fast rod pathways.

## Introduction

Congenital stationary night blindness (CSNB) is a clinically and genetically heterogenous disorder and may follow autosomal dominant, autosomal recessive, or X-linked recessive patterns of inheritance. The clinical features may vary between subtypes, but affected individuals often complain of nyctalopia since childhood and commonly present with nystagmus, reduced visual acuity, strabismus, and myopic refractive error [1]. Although commonly referred to as a stationary disease with a normal-looking retina, except for myopic changes, there are reports of progressive vision loss [2–4], optic atrophy [2], and retinal pigment epithelial abnormalities [2,4] in CSNB. Electroretinography (ERG) aids in diagnosing and classifying CSNB. Broadly, two distinct electrophysiological patterns are recognized; the Riggs type of ERG [5] is observed when the deficit involves phototransduction, and the Schubert-Bornschein type of ERG [6] is observed when the deficit is post-phototransduction. The cardinal feature of Schubert-Bornschein ERG is an electronegative response to standard flash (normal a-wave, reduced b-wave and b:a ratio <1.0) under scotopic conditions. Miyake et al. [1] further classified patients with the Schubert-Bornschein type of ERG into complete and incomplete phenotypes based on the absence or presence of rod function, respectively, to dim white light stimulation and noted that involvement of the cone system also differed between the groups. Mutations in the α1 subunit of the L-type voltage gated Ca^2+^ channel gene (*CACNA1F*; OMIM *300110) is associated with X-linked incomplete CSNB (CSNB2A; OMIM #300071) [7,8]. Expressed predominantly in the outer and inner nuclear layers of the retina [7], these channels support Ca^2+^ influx under relatively depolarized conditions, which is necessary for tonic glutamate release from rod and cone photoreceptors [9,10]. Mouse and rat models of *Cacna1f* also confirm the deficit to be in the transmission of the signal from photoreceptors to second-order neurons [7,11].

Åland island eye disease (AIED; OMIM #300600) or Forsius-Eriksson syndrome is an X-linked disorder first described in Norwegian descendants on the Åland Islands in the Sea of Bothnia [12]. Affected males have nystagmus, myopia, reduced visual acuity, red-green color vision deficits, iris trans-illumination defects, foveal hypoplasia, and a blonde fundus without evidence of chiasmal misrouting [12,13]. Although an incomplete type of Schubert-Bornschein ERG was not observed originally in AIED, this has subsequently been described [14]. Two reports of AIED pedigrees with mutations identified in *CACNA1F* have been published [15,16]; hence, CSNB2A and AIED are considered to be allelic variants.

The present study describes, for the first time, two affected members of the same pedigree with distinct features of AIED in one and of incomplete CSNB (CSNB2A) in the other, both associated with a novel p.G603R missense mutation in the *CACNA1F* gene. This study also presents novel electrophysiology data describing the slow and fast rod pathway activity in *CACNA1F*- related disease.

## Methods

### Clinical evaluation

**Figure 1:**
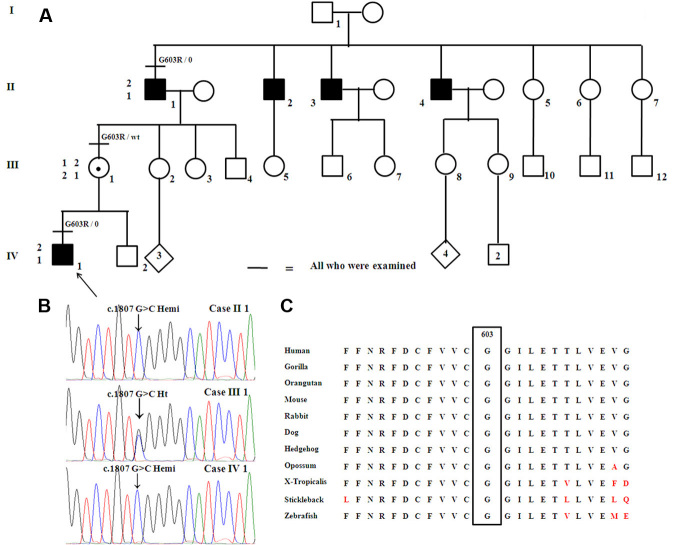
The family pedigree, chromatogram, and amino-acid conservation map are shown. **A**: Pedigree of the family studied demonstrating the X-linked inheritance pattern. The haplotypes for the polymorphic DNA markers flanking *CACNA1F* (DXS1003 and DXS7132; proximal and distal, respectively) in the three family members are also included. **B**: Chromatogram of the portion of *CACNA1F* exon 14 showing the c.1807G>C hemizygous change in cases II 1 and IV 1. Case III 1 is a female carrier and had no manifestations. **C**: Amino acid conservation map across species demonstrating that glycine at position 603 is conserved lower down to sticklebacks and zebrafish.

The study protocol was approved by the Research Ethics Board at The Hospital for Sick Children and adhered to the Tenets of the Declaration of Helsinki. Three members (including two affected males) of a Caucasian family were evaluated (Figure 1A).

All three subjects underwent ophthalmologic evaluation that included best corrected visual acuity (BCVA) and color vision assessment (Hardy-Rand-Rittler plates), contrast sensitivity measurement (Pelli-Robson charts), slit-lamp evaluation, dilated retinal examination, fundus autofluorescence imaging (Visucam; Carl Zeiss Meditec, Dublin, CA), and spectral domain optical coherence tomography (SD-OCT, Cirrus; Carl Zeiss Meditec). Goldmann visual field analysis was performed on the two affected subjects.

### Electrophysiology

Full-field ERG incorporating the International Society for Clinical Electrophysiology of Vision (ISCEV) Standards [17] was performed on the two affected individuals. The four ERG responses evaluated include dim light scotopic response [flash intensity, 0.01 cd-seconds per square meter (cd.s.m^−2^)], combined rod-cone response to standard flash (2.29 cd.s.m^−2^), single flash photopic response (2.29 cd.s.m^−2^), and 30 Hz flicker response (2.29 cd.s.m^−2^). Multichannel flash visual evoked potential with active electrodes placed at scalp positions Oz, O_1_, O_2_, PO_7_, and PO_8_ was performed on the proband (case IV 1) [18,19]. Additionally, a series of 15 Hz flicker ERGs was performed under scotopic conditions to study the slow (rod → rod On bipolar cell → AII amacrine) and fast (rod → rod-cone gap junction → cone On bipolar cell) rod pathways [20]. Serial 15 Hz stimuli incrementing in intensity from −3.40 log scotopic troland-second (scot td-sec) to +0.35 log scot td-sec (steps of 0.1–0.3 log units) were presented using a Ganzfield bowl (Espion, Diagnosys LLC, Boston, MA) using equivalent techniques as reported previously [21]. Stimuli were created by combining colored light-emitting diodes to generate white flashes of 4 ms duration. At lower light intensities (<–1.0 log scot td-sec), 100 trials in length (each trial lasted 200 ms and was composed of 3 flashes) were recorded and averaged; this was reduced to 30 trials at higher flash intensities to minimize patient discomfort. In adults with normal retinal function, slow rod pathway activity predominates at lower intensities (<–1.97 log scot td-sec), and fast rod pathway activity predominates at higher intensities (>–1.37 log scot td-sec) [21,22]; however, inter-laboratory variations have been reported [23,24].

### Genetic analysis

Haplotype analysis was performed on all three members of the family using polymorphic microsatellite DNA markers flanking *CACNA1F* (DXS1003 and DXS7132; PCR conditions for both: 1.5 mM MgCl_2_; annealing temperature: 57 °C; cycles: 35). All exons and exon/intron boundaries of *CACNA1F* and nyctalopin (*NYX*) genes were sequenced in the proband (case IV 1) at the University of Colorado Denver DNA diagnostic laboratory. The *CACNA1F* exon 14 harboring the variant of interest in all three subjects was amplified and sequenced at the Héon laboratory (PCR conditions: 1.5 mM MgCl_2_, 1× Q Solution (Qiagen GmbH, Hilden, Germany); annealing temperature: 62.3 °C; cycles: 36; forward primer: 5′-GCC TGA ATA CCG AGC ACA TT-3′; reverse primer: 5′-TGT TGA GGC TGT TTG AGG-3′). The allele frequency of the novel change (c.1807G>C) was evaluated in 360 ethnically matched control chromosomes with amplification-refractory mutation system assay designed for the mutant C allele (PCR conditions: 1.0 mM MgCl_2,_ 1× Q solution; annealing temperature: 60.7 °C; cycles: 38; forward primer: 5′-TGA CTG CTT TGT GGT CAG TC-3′; reverse primer: same as above).

## Results

The inheritance pattern of the phenotype was determined to be X-linked (Figure 1A). The detailed phenotypic and genotypic characteristics are described below.

### Phenotypic characteristics

#### Proband (case IV 1)

**Figure 2:**
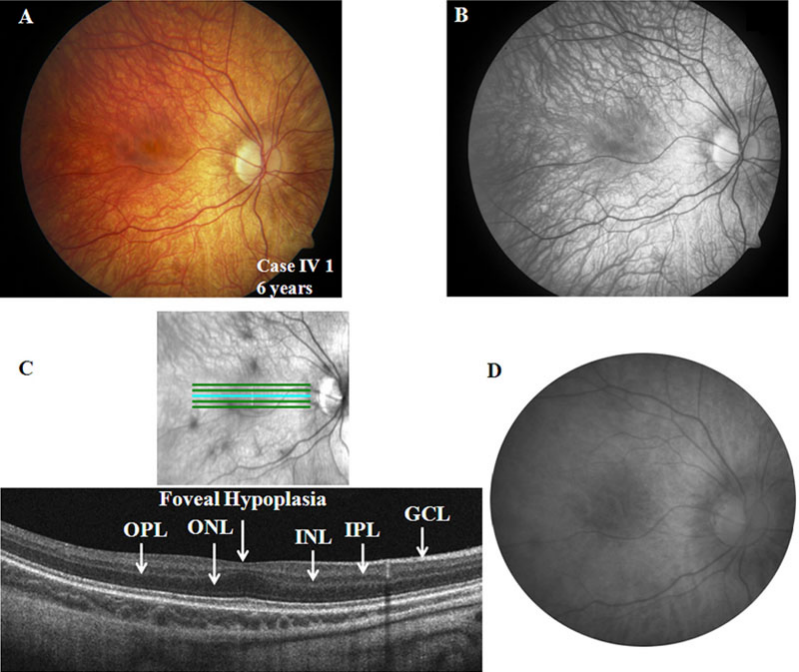
Ocular phenotypic characteristics of the proband (case IV 1; only right eye details shown). **A**, **B**: Fundus photograph and red free image showing hypopigmented fundus and foveal hypoplasia, respectively. **C**: Spectral domain optical coherence tomography demonstrating widening of the outer nuclear layer (ONL) at the fovea. The layers inner to ONL, including the outer plexiform layer (OPL), the inner nuclear layer (INL), and the inner plexiform layer (IPL), are all present at the fovea consistent with foveal hypoplasia. The ganglion cell layer (GCL) is also labeled. **D**: Fundus autofluorescence image of the posterior pole revealed normal autofluorescence.

A six-year-old male child presented with a history suggestive of nyctalopia and photophobia from childhood. Parents have not noted any nystagmus. The symptoms have remained stationary since birth. The family history is significant for similar symptoms in the maternal grandfather’s generation (Figure 1A). The child was born blond but now tans easily. On examination, he was orthophoric and had minimal nystagmus in the right eye. He had high myopic refraction, and his BCVA was 20/50 and 20/40 in the right and left eyes, respectively. Color vision testing showed a mild deficit in the red-green axis. His log contrast sensitivity was 1.65 in each eye. The iris did not show any trans-illumination defects. The fundus was markedly hypopigmented and showed foveal hypoplasia (Figure 2A,B). The SD-OCT demonstrated a shallow foveal pit, the presence of inner plexiform and inner nuclear layers at the fovea, and a widened outer nuclear layer consistent with Grade 1 foveal hypoplasia (Figure 2C). The autofluorescence was normal in the posterior pole (Figure 2D).The Goldman visual fields showed normal boundaries to I4e and III4e stimulus targets.

**Figure 3:**
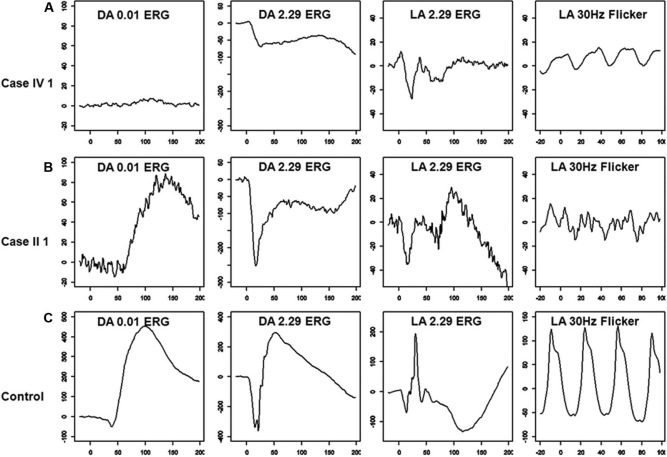
Full-field electroretinogram (ERG) characteristics in case IV 1 (**A**), case II 1 (**B**), and a control subject (**C**) are presented. Dim light scotopic response (dark adapted [DA] ERG to 0.01 cd-seconds per square meter–DA 0.01 ERG) showed severe b-wave reduction in case IV 1 (3A) and was at the lower limit of normal in case II 1 (**B**). Both cases showed electronegative configuration to combined rod-cone response (DA ERG to 2.29 cd-seconds per square meter – DA 2.29 ERG; **A** and **B**), and case IV 1 showed additional a-wave involvement. Single flash photopic response (light adapted (LA) ERG to 2.29 cd-seconds per square meter–LA 2.29 ERG) showed severe reduction and delay of the a- and b-waves with reduced b/a ratio in both subjects (**A** and **B**). The 30 Hz flicker response (LA 30 Hz flicker) showed severely reduced and delayed b-waves in both cases (**A** and **B**). Please note that the scale on the y-axis is different for the cases and the control.

**Figure 4:**
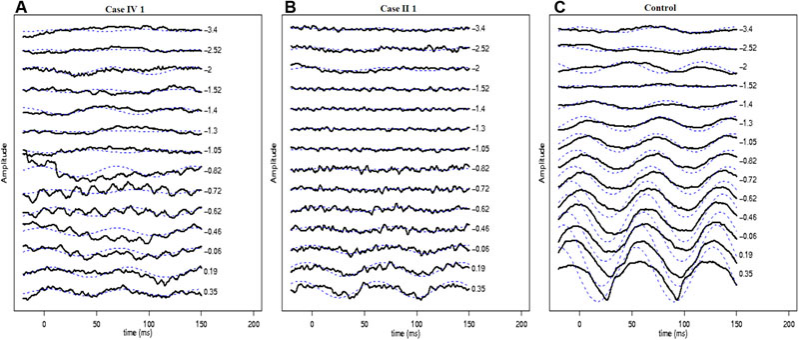
Scotopic 15 Hz flicker electroretinogram characteristics in the proband (**A**), case II 1 (**B**), and a control subject (**C**) are presented. Various intensities between −3.4 log scot td-sec and +0.35 log scot td-sec were performed. Dashed lines show the response filtered using a simple Fourier domain, bandpass filter (13–18 Hz). In the control panel (**C**), the rod system activity observed between −3.40 log scot td-sec and −2.0 log scot td-sec intensities originates from the slow rod pathway. At an intensity of −1.52 log scot td-sec, there is no discernable rod activity denoting neutralization of activity between the slow and fast rod pathways (**C**). At intensities of −1.30 log scot td-sec, the rod system activity reappears (which is out of phase to the response seen at −2.0 log scot td-sec and originates in the fast rod pathways), which progressively increases in amplitude and shows progressive advancement in phase (**C**). In case IV 1 and case II 1, the rod activity is discernable only at intensities higher than +0.19 log scot td-sec and −0.62 log scot td-sec, respectively (figures **A** and **B**, respectively). This suggests that only residual fast rod pathway activity is seen in *CACNA1F*-related disease.

Full-field ERG showed severely reduced dim light scotopic response, electronegative configuration of combined rod-cone response, severely reduced and delayed single flash photopic response, and markedly reduced 30 Hz flicker response (Figure 3A; compare with Figure 3C traces of a control subject). The scotopic 15 Hz flicker showed no discernable rod activity between intensities of −3.40 log scot td-sec and −0.46 log scot td-sec (Figure 4A). The rod activity was demonstrable first at an intensity of +0.19 log scot td-sec and was noted at higher intensities, suggesting residual activity in the fast rod pathways (Figure 4A; compare with Figure 4C traces of a control subject). Multichannel visual evoked potential showed no inter-hemispheric difference to monocular stimulation (Pearson’s correlate=0.8), thus ruling out the presence of chiasmal misrouting. The phenotypic characteristics of the proband were consistent with AIED with an incomplete Schubert-Bornschein type of ERG.

#### Case II 1

**Figure 5:**
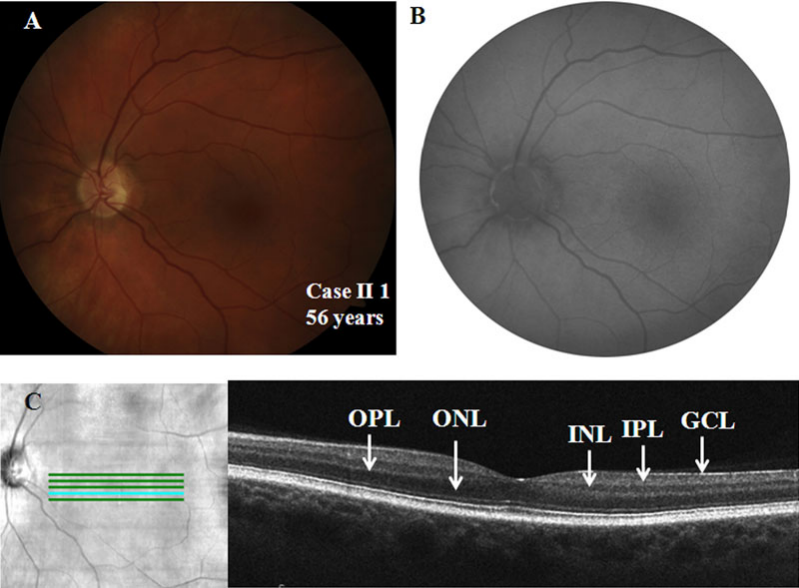
Ocular phenotypic characteristics of case II 1 (only left eye details are shown) **A**: Fundus photograph showing normal pigmentation and dull foveal reflex. **B**: Fundus autofluorescence image of the posterior pole revealed normal autofluorescence. **C**: Spectral domain optical coherence tomography of the left eye revealed normal foveal contour and central retinal thickness. All relevant outer and inner retinal layers have been labeled: the outer nuclear layer (ONL), the outer plexiform layer (OPL), the inner nuclear layer (INL), the inner plexiform layer (IPL), and the ganglion cell layer (GCL).

The 56-year-old grandfather of the proband had complaints of non-progressive nyctalopia since childhood but had no symptoms of photophobia. On examination, he was orthophoric with a BCVA of 20/50 in each eye, and he had no significant refractive error. His color vision and contrast sensitivity (1.65 log units) were normal in each eye. The anterior segment evaluation was normal. The fundus showed normal pigmentation; however, the foveal reflex was dull (Figure 5A). The fundus autofluorescence was normal (Figure 5B), and Goldmann visual field testing showed normal boundaries to I4e and III4e stimulus targets. The SD-OCT showed a normal foveal pit and central retinal thickness (Figure 5C).

Full-field ERG showed borderline normal dim light scotopic response, electronegative configuration of combined rod-cone response, severely reduced and delayed single flash photopic response, and markedly reduced 30 Hz flicker response (Figure 3B; compare with Figure 3C traces of a control subject). The scotopic 15 Hz flicker showed no discernable rod activity between intensities of −3.40 log scot td-sec and −0.72 log scot td-sec (Figure 4B). Rod activity was first noted at −0.62 log scot td-sec and was subsequently present at all higher flash intensities, suggesting residual activity in the fast rod pathways (Figure 4B; compare with Figure 4C traces of a control subject). The phenotypic characteristics of the subject were consistent with incomplete CSNB (CSNB2A).

#### Case III 1

The 36-year-old mother of the proband was asymptomatic. On examination, she had exophoria, and her BCVA was 20/20 in each eye. Her color vision and contrast sensitivity were normal. Fundus evaluation, autofluorescence, and SD-OCT were unremarkable.

### Genetic results

Haplotype analysis of the region adjoining *CACNA1F* supported the role of the gene in this family (Figure 1A). A novel hemizygous missense mutation c.1807G>C (NM_005183.2; p.G603R) was identified in the proband and the change segregated with the disease phenotype (Figure 1B). The mutation is located on the S3 helical trans-membrane domain repeat II of the *CACNA1F* gene (*CACNA1F* encodes for the α1 subunit of a L-type Ca^2+^ channel that contains four repeat domains, each of which has six transmembrane domains (S1–S6); NP_005174.2). The novel c.1807G>C change was not seen in 360 control chromosomes, and the glycine at position 603 is conserved in lower vertebrates such as stickleback and zebrafish (UCSC Conservation track; Figure 1C). The p.G603R was predicted to be possibly damaging by Polyphen v. 2.09 with a PSIC score difference of 1.525. The mutation was predicted also to affect protein function by SIFT with a score of 0.00. No mutations were identified in *NYX*.

## Discussion

This is the first report in a molecularly confirmed *CACNA1F* pedigree with two affected members showing distinct classical phenotypes of AIED and CSNB2A, respectively. Since the association of CSNB2A with *CACNA1F* [7,8], few instances of deletions, either in *CACNA1F* (two pedigrees) or in the regions of Xp21 (one case), were reported with the classical AIED phenotype [15,16,25], suggesting AIED and CSNB2A are allelic with specific mutations causing each particular phenotype. The novel p.G603R mutation identified in this family confirms AIED and CSNB2A are allelic. Furthermore, the phenotypic variability observed in this family suggests the presence of other modifiers (probably genetic) yet to be identified in *CACNA1F-* related disease. This finding implies that the phenotypic variability observed between CSNB2A and AIED is not a consequence of intragenic allelic heterogeneity alone. Before the *CACNA1F* gene was discovered, a pedigree of X-linked incomplete CSNB was reported with some cases showing hypopigmented fundus (with no foveal hypoplasia), supporting this concept [26]. Further, Boycott et al. [27] showed the absence of one of the three major features of CSNB (nyctalopia, myopia, and nystagmus) in approximately three quarters of a large cohort of patients carrying the same *CACNA1F* mutation, which also suggests the presence of modifying factors.

Although AIED and CSNB2A have many overlapping clinical features, the former has additional features such as progressive myopia, dyschromatopsia, iris trans-illumination defects, and foveal hypoplasia [1,12]. The AIED phenotype in this study demonstrated all the features except for iris trans-illumination deficits. Animal models demonstrate abnormal ribbon synapses in photoreceptors and abnormal synaptogenesis in that a portion of second-order neurons and horizontal cells synapse in the outer nuclear layer or retinal pigment epithelium rather than in the outer plexiform layer [9–11,28–30]. This may affect centrifugal migration of the inner retinal layers at the fovea, which happens in human eyes during development [from 24 weeks gestation to 15 months postpartum [31]] and may result in foveal hypoplasia seen in the AIED phenotype. The SD-OCT demonstrated a shallow foveal pit, persistence of all the inner retinal layers, and widening of the outer nuclear layer in the AIED phenotype, consistent with Grade 1 foveal hypoplasia [32]. The autofluorescence in the posterior pole was normal in the two affected cases, suggesting a normal lipofuscin level at the retinal pigment epithelium in *CACNA1F-*related disease. There was no evidence of visual field constriction in either affected individual as observed previously [33]. Rare progressive phenotypes with central field scotomas also have been reported [2].

The AIED and CSNB2A phenotypes in this study revealed an incomplete Schubert-Bornschein type of ERG, as previously reported [2,14]. Interestingly, the individual with the CSNB2A phenotype, who was much older (the grandfather of the proband), showed milder scotopic ERG abnormalities. Both individuals showed severe and identical photopic ERG abnormalities. It is tempting to relate the severe abnormality in ERG directly to the AIED phenotype, but the proband also had high myopia, which is known to affect ERG amplitude [34,35], thus precluding an accurate conclusion. This is the first report to assess scotopic 15 Hz flicker ERGs in *CACNA1F* phenotypes. There was no rod activity from the slow rod pathways in either affected individual. The rod activity appeared at intensities of +0.19 and −0.62 log scot td-sec in the AIED and CSNB2A phenotypes, respectively. This reflects residual activity originating in the fast rod pathways in *CACNA1F*-related disease. The appearance of fast rod activity at a higher intensity in the AIED phenotype is probably a consequence of a more severe scotopic ERG abnormality observed in this subject (see above). Further, cone activity under scotopic conditions is known not to appear until intensities of +0.75 log scot td-sec [36], thus ruling out a cone contribution to the observed scotopic 15 Hz flicker responses in this study. Previously, two genes associated with complete CSNB (*NYX* and transient receptor potential cation channel, subfamily M, member 1 [*TRPM1*]) [21,24] have been associated with identical scotopic 15 Hz ERG findings as those now found in *CACNA1F-*related disease. On the contrary, calcium binding protein 4 (*CABP4*) related autosomal recessive incomplete CSNB shows preservation of slow rod pathways and severely attenuated or absent fast rod pathways [23]. Hence, the scotopic 15 Hz flicker ERG could be useful in differentiating between *CACNA1F*- and *CABP4*-related incomplete CSNB if the inheritance pattern is unknown.

To conclude, this pedigree with a novel p.G603R mutation in *CACNA1F* confirms that CSNB2A and AIED are indeed allelic variants, but also illustrates that the two are not mutation specific. An incomplete Schubert-Bornschein type of ERG was seen in both phenotypes, and the residual rod activity demonstrable in *CACNA1F*-related disease arises predominantly from the fast rod pathways. The underlying modifying factor responsible for the phenotypic variability remains to be elucidated.
